# THE POWERFUL TOOLS FOR CAREGIVERS TOOLKIT: ENHANCING SELF-CARE AND COMMUNICATION FOR QUALITY CARE

**DOI:** 10.1093/geroni/igae098.1778

**Published:** 2024-12-31

**Authors:** JeongEun Lee, Joseph Svec, Eunbea Kim, Yulri Kim

**Affiliations:** Iowa State University, Ames, Iowa, United States; St. Joseph’s University, Patchogue, New York, United States; Iowa State University, Ames, Iowa, United States; Iowa State University, Ames, Iowa, United States

## Abstract

The Powerful Tools for Caregiver (PTC) program, an evidence-based program, has proven to enhance caregivers’ self-care and communication skills. Prior studies substantiate its efficacy in elevating caregiver competence and promoting self-care practices. This study delves into secondary outcomes, emphasizing perceived relationship quality with care recipients with dementia. We examined responses from 340 caregivers engaged in the Powerful Tools for Caregivers program delivered with both in-person and remote module. Utilizing pre- and post-surveys, we conducted MANCOVA and multiple regression analyses. Findings revealed that caregivers of dementia reported improved self-care, increasing communication competence, and enhancing positive role perceptions. A majority caregivers reported significant increase in allocating time for personal well-being (average 1.24 hours per week) and discovering effective solutions to caregiving challenges using action plans. Moreover, caregivers allocating more time to self-care reported delivering higher-quality care and experiencing improved relationship qualities with care recipients. Notably, these positive changes were attributed to enhanced communication strategies and positive perspectives adopted by caregivers. The findings underscore the pivotal role of self-care and effective communication in augmenting the quality of care provided to recipients. Comparing the module of the delivery, we did not find any significance, confirming parallel effectiveness of the program. In conclusion, this research illuminates the significance of prioritizing self-care and honing communication skills in improving the wellbeing of both caregivers and care recipients with dementia. The paper concludes by outlining implications derived from the findings and proposes avenues for future research to further enrich our understanding of caregiver support programs.

